# Predictive performance of six mortality risk scores and the development of a novel model in a prospective cohort of patients undergoing valve surgery secondary to rheumatic fever

**DOI:** 10.1371/journal.pone.0199277

**Published:** 2018-07-06

**Authors:** Omar A. V. Mejia, Manuel J. Antunes, Maxim Goncharov, Luís R. P. Dallan, Elinthon Veronese, Gisele A. Lapenna, Luiz A. F. Lisboa, Luís A. O. Dallan, Carlos M. A. Brandão, Jorge Zubelli, Flávio Tarasoutchi, Pablo M. A. Pomerantzeff, Fabio B. Jatene

**Affiliations:** 1 Department of Thoracic and Cardiovascular Surgery, Heart Institute–University of São Paulo Medical Center, São Paulo, Brazil; 2 Center of Cardiothoracic Surgery, University Hospital and Faculty of Medicine, Coimbra, Portugal; 3 National Institute for Pure and Applied Mathematics, Rio de Janeiro, RJ, Brazil; 4 Department of the Clinical Unit of Heart Valve Diseases, Heart Institute–University of São Paulo Medical Center, São Paulo, Brazil; Boston University, UNITED STATES

## Abstract

**Background:**

Mortality prediction after cardiac procedures is an essential tool in clinical decision making. Although rheumatic cardiac disease remains a major cause of heart surgery in the world no previous study validated risk scores in a sample exclusively with this condition.

**Objectives:**

Develop a novel predictive model focused on mortality prediction among patients undergoing cardiac surgery secondary to rheumatic valve conditions.

**Methods:**

We conducted prospective consecutive all-comers patients with rheumatic heart disease (RHD) referred for surgical treatment of valve disease between May 2010 and July of 2015. Risk scores for hospital mortality were calculated using the 2000 Bernstein-Parsonnet, EuroSCORE II, InsCor, AmblerSCORE, GuaragnaSCORE, and the New York SCORE. In addition, we developed the rheumatic heart valve surgery score (RheSCORE).

**Results:**

A total of 2,919 RHD patients underwent heart valve surgery. After evaluating 13 different models, the top performing areas under the curve were achieved using Random Forest (0.982) and Neural Network (0.952). Most influential predictors across all models included left atrium size, high creatinine values, a tricuspid procedure, reoperation and pulmonary hypertension. Areas under the curve for previously developed scores were all below the performance for the RheSCORE model: 2000 Bernstein-Parsonnet (0.876), EuroSCORE II (0.857), InsCor (0.835), Ambler (0.831), Guaragna (0.816) and the New York score (0.834). A web application is presented where researchers and providers can calculate predicted mortality based on the RheSCORE.

**Conclusions:**

The RheSCORE model outperformed pre-existing scores in a sample of patients with rheumatic cardiac disease.

## Introduction

Approximately 80% of countries worldwide present with rheumatic fever (RF) and with one of its most prevalent complications, the rheumatic heart disease (RHD). People presenting advanced RHD without access to cardiac surgery die [1].

An improvement in our ability to predict who the best surgical candidates might be can partially account for recent improvements in mortality rates after cardiac procedures. This prediction is frequently accomplished through risk scores. As a consequence, numerous risk scores have been developed over time [2–5]. Although the widespread use of risk scores is deemed to be a sign of improvement in our clinical decision support system, clinicians often fail to notice that the performance of a given risk score only remains adequate under certain conditions. For example, if the sample which validated the risk score was different from the patient population where it is being applied, then prediction performance could be compromised, ultimately resulting in misleading clinical decisions.

Around the world, there are over 15 million people with RHD accompanied by 300,000 new cases per year and over 200,000 annual deaths [6]. The public health system of Brazil spends over 90 million dollars a year to treat patients with RF and RHD, thus the creation of a task force for the prevention [7] and improvement of quality initiatives for the surgical treatment of patients with RHD such as repair rather than replacement of diseased valves because of renowned consequences [8]. However, RHD damages the valve leaflets and the subvalvular apparatus, making the repair more difficult. For these reasons, proper risk assessment for purposes of informed consent and the determination of current treatment in these patients is important because the traditional risk scores often emerge from non-rheumatic populations. Given that most risk scores to date were developed and validated mostly among patients in developed countries, it is questionable whether their predictive performance would still be optimal when applied to rheumatic patients. Unfortunately, we are not aware of any previous scores validated among a large, prospective sample exclusively composed of patients with a diagnosis of rheumatic valve disease.

In the face of this gap in the literature, our study aimed to evaluate the predictive performance of six different risk scores: the 2000 Bernstein-Parsonnet, EuroSCORE II, InsCor, Ambler, Guaragna and the New York scores. In addition, we developed the RheSCORE model, optimized for mortality risk prediction among patients with rheumatic valve disease. It was our hypothesis that a prediction model specifically designed to be used among patients with rheumatic valve disease would outperform previously existing scores.

## Materials and methods

### Study design

This study is a prospective consecutive all-comers cohort of patients referred to the Department of Thoracic and Cardiovascular Surgery, Heart Institute–University of São Paulo Medical Center, São Paulo, Brazil.

### Study population

Between May 2010 and July of 2015, a total of 2,919 consecutive all-comers patients with RHD referred for surgical treatment of valve disease. Symptomatic RHD was characterized by 2004 World Health Organization criteria for the diagnosis of first onset, recurrence and chronic RHD (modified Jones criteria) and transthoracic echocardiography. Patients were excluded from the study if the primary diagnosis of the valvular disease was not RHD or if they were undergoing associated procedures such as myocardial revascularization, ASD closure, thoracic aorta procedures, etc. The ESC/EACTS 2012 guideline was used for surgical indication of valvular heart disease. All data were extracted from the general prospective institutional register (Si3) and stored in compliance with institutional security and privacy governance rules. To ensure data accuracy, quality checks were performed over time by the postgraduate student and the supervisors (authors).

### Predicting variables

Our choice of risk scores was defined by a consensus among participating surgeons, decisions being made on the basis of their methodology and popularity in the literature as well as applicability (Table 1). Clinical and laboratory-related variables for the 2000 Bernstein Parsonnet [2], EuroSCORE II [9], InsCor [5], Ambler [10], New York [11] and Guaragna [12] (Table 2) were prospectively collected and subsequently scored according to the criteria and definitions stipulated by their developers as well as for our new proposed model, RheSCORE.

**Table 1:** Design of selected risk scores.

	Conditions	Outcome	Setting	Sample characteristics	Score validation	Predictive model
**2000 Bernstein Parsonnet (2000)**	coronary artery bypass graft surgery + Valve	In-hospital Mortality	Multicenter (10 centers)/New Jersey	Retrospective; 8593 patients	Retrospective: 2110 patients	Logistic
**EuroSCORE II (2011)**	coronary artery bypass graft surgery + Valve	Hospital mortality	Multicenter (154 centers) / European & Non-European	Prospective:22381 patients	Prospective:5553 patients	Logistic
**InsCor (2012)**	coronary artery bypass graft surgery + Valve	In-hospital Mortality	Single center/Brazil	Prospective:2000 patients	Prospective:1000 patients	Logistic with bootstrap
**NewYorkscore(2007)**	Valve	Mortality	Multicenter (New York Database)/USA	Retrospective:10702 patients	Retrospective:9662 patients	Logistic
**Ambler score (2005)**	Valve (coronary artery bypass graft surgery)	In-hospital Mortality	Multicenter (Great Britain and Ireland Database)/England	Prospective:16679 patients	Prospective: 16160 patients	Logistic
**Guaragna score (2010)**	Valve	In-hospital Mortality	Single center/Brazil	Retrospective:699 patients	Retrospective: 387 patients.	Logistic

**Table 2:** Variables included in each risk score.

Score variables	2000 BP	EuroSCO-RE II	InsCor	Amblerscore	New York score	Guaragnascore	RheSCORE
Patient data							
Age	x	x	x	x	x	x	x
Gender	x	x	x	x	x	x	
Weight	x *			x			
Unstable angina		x					
Left-sided disease	x						
Active endocarditis	x	x			x		
Systemic hypertension	x			x		
Pulmonary hypertension	x	x				x	x
Left ventricular aneurysm	x						
Low left ventricular ejection fraction	x	x	x	x		x	x
Myocardial infarction	x	x	x		x		
High class NYHA* functional classification		x				x	
Post infarction ventricular septal defect	x						
Ventricular tachycardia/fibrillation	x	x	x	x			
Cardiac resuscitation		x					
Atrial fibrillation				x			x
Enlarged left atrium							x
Asthma	x	x					
Chronic obstructive pulmonary disease **	x	x			x		
Dialysis	x			x	x	x	
Creatinine		x	x	x	x	x	x
Acute renal failure	x	x					
Creatinine clearance		x					
Diabetes	x	x		x			
Liver disease	x				x		
Transitory ischemic attack	x	x				
Idiopathic thrombocytopenic purpura	x						
Disability affecting mobility		x					
Blood products refused	x						
Percutaneous transluminal coronary angioplasty failure	x						
Substance abuse	x						
Peripheral artery disease	x	x			x		
History of vascular surgery	x	x					
Carotid disease	x	x					
Preop ventilation	x	x	x				x
Preop intra-aortic balloon pump	x	x	x				
Preop inotropes		x	x		x *		x
Preop resuscitation		x	x				
Preop cardiogenic shock	x		x		x		x
Combined coronary artery bypass graft surgery + valve surgery	x	x	x	x		x	
Multiple valve procedure		x		x	x		
Urgent/emergency/salvage procedure		x		x		x	
Reoperation	x	x	x	x	x		X
Aortic valve surgery		x	x	x	x		
Tricuspid valve surgery	x	x	x	x			
Mitral valve surgery	x	x		x	x *		

* NYHA (New York Heart Association)

** COPD (Chronic obstructive pulmonary disease)

### Outcome variables

The outcome variable of interest was hospital mortality, defined as death in the hospital or within 30 days of cardiac surgery.

### Data analysis

We followed international reporting guidelines as well as an expert recommendation in our modeling strategy. We started the analysis by performing a graphical exploratory analysis evaluating the frequency, percentage and near-zero variance for categorical variables, distribution for numeric variables, and missing values and patterns across all variables. In addition, a Maximal Information Nonparametric Exploration algorithm was run to guide bivariate plot inspection. Feature engineering then proceeded by attempting variable transformations and dummy coding for variables with distributions that were not normal at inspection, variable recategorization or removal for near-zero variation, and different imputation algorithms for variables with missing values. We modeled hospital mortality as an outcome variable. To train and test our models, we used a five-fold model validation. The RheSCORE modeling processed involved Random Forests, Quadratic Discriminant Analysis, Generalized Linear Model with binomial distribution family, Linear Discriminant Analysis, Partial Least Squares, Penalized Logistic Regression, Nearest Shrunken Centroids, Mixture Discriminant Analysis, Neural Network, Flexible Discriminant Analysis, Support Vector Machines with Radial Basis Function Kernel, k-Nearest Neighbors and Naive Bayes. Model tuning was performed using the parameters listed in Table 3.

**Table 3:** Models and corresponding tuning parameters.

Model	Tuning parameters
Random Forests	Number of Randomly Selected Predictors
Quadratic Discriminant Analysis	No tuning parameters
GLM binomial distribution family	No tuning parameters
Linear Discriminant Analysis	Number of Discriminant Functions
Partial Least Squares	Number of Components
Penalized Logistic Regression	L2 penalty and complexity parameter
Nearest Shrunken Centroids	Shrinkage threshold
Mixture Discriminant Analysis	Number of subclasses per class
Neural Network	Number of hidden units, weight decay
Flexible Discriminant Analysis	Product degree and number of terms
Support Vector Machines with Radial Basis Function Kernel	Sigma, cost, weight
k-Nearest Neighbors	Maximum number of neighbors, distance, kernel
Naive Bayes	Laplace correction, distribution type

Comparison across models was performed using metrics for the area under the curve, sensitivity, specificity, Kappa as well as positive and negative predictive values. All calculations were performed using the statistical language R, including packages ggplot2, caret, rmarkdown, vcd, randomforest, MASS, glmnet, mda, pROC, corrplot, and tabplot. Finally, total scores for the 2000 Bernstein-Parsonnet, EuroSCORE II, InsCor, Ambler, Guaragna, and the New York score were used to predict mortality using logistic regression models under identical validation criteria used for the RheSCORE model.

### Ethical approval

This study is part of the project: “Mortality prediction in coronary bypass surgery and/or heart valve surgery at InCor: Validation of two external risk models and comparison to the locally developed model (InsCor)” approved with the number 1063/07 (SDC: 3073/07/148) by the Ethics Committee of the Heart Institute of the Hospital das Clinicas, Medicine School, University of São Paulo, Brazil. Because our study used a pre-established database, the use of informed consent forms was waived.

### Companion web site

A companion site was designed to contain additional, up-to-date information on the data set, model as well as a Web Application that can perform mortality predictions based on individual patient characteristics. The application was developed using the Shiny framework.

## Results

**Fig 1:**
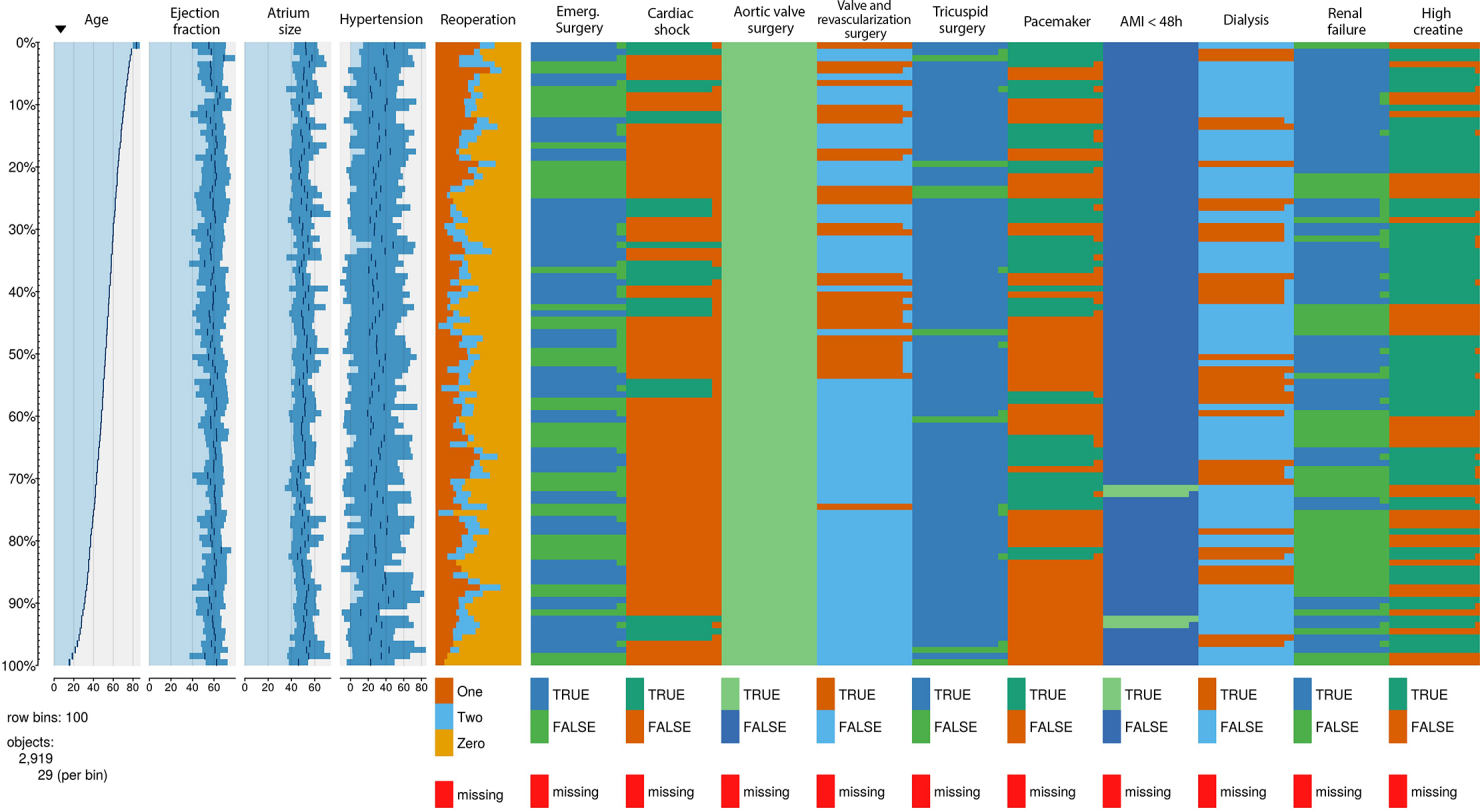
A. Distribution of the sampling variables. Age: age at surgery; eject_fracti: ejection fraction; atrium_size: left atrial size; hypertensio: pulmonary hypertension; reoperation: number of previous cardiac surgeries; emergency: emergency surgery; cardiac_sh: cardiogenic shock; aortic valve: aortic valve surgery B. Distribution of sampling variables. Valve_revasc: heart valve surgery and CABG; tricuspid: tricuspid valve surgery; pacemaker: pacemaker dependency; ami48h: acute myocardial infarction 48h after cardiac surgery; dialysis: renal replacement therapy after cardiac surgery; renal_failure: acute kidney injury after cardiac surgery; high_creatinine: creatinine levels higher than 2mg/dl.

A total of 2,919 RHD patients underwent heart valve surgery. A hospital mortality rate of 3,51% was recorded for the entire population. Mortality rates associated with aortic, mitral and tricuspid surgery were 2,43%, 3,85%, and 7,25% respectively. Our study sample mostly composed of patients above the age of 50 years, with over 40% having undergone at least one previous surgical procedure, and with the aortic valve being the most common valve location. A number of baseline variables were significantly different for the group of patients who died and those who did not, including lower ejection fraction, pulmonary hypertension, reoperations, emergency, cardiogenic shock, aortic valve surgery, tricuspid valve surgery, renal failure, dialysis and high creatinine values (Table 4). A more pronounced heterogeneity demonstrated by increased variability was observed among variables such as pulmonary hypertension, reoperation and aortic and tricuspid valve surgery procedures (Fig 1A and 1B). In these graphics, all variables are presented in relation to the distribution of age (left-most column).

**Table 4:** Characteristics of sample.

	Survival (N = 2820)	Death (N = 99)	p
Age	51.2 ± 14.9	54.4 ± 17.4	0.076
Ejection fraction	59.4 ± 11.8	52.7 ± 15.7	0.000
Left atrial size	50.4 ± 10.0	63.4 ± 16.9	0.000
Pulmonary Hypertension	30.0 ± 31.7	43.1 ± 35.2	0.000
Reoperation			0.000
- Zero	1644 (58.3%)	30 (30.3%)	
- First	780 (27.7%)	33 (33.3%)	
- Second	396 (14.0%)	36 (36.4%)	
Emergency			0.000
- No	2724 (96.6%)	81 (81.8%)	
- Yes	96 (3.4%)	18 (18.2%)	
Cardiogenic shock			0.000
- No	2796 (99.1%)	87 (87.9%)	
- Yes	24 (0.9%)	12 (12.1%)	
Aortic valve surgery			0.005
- No	1464 (51.9%)	66 (66.7%)	
- Yes	1356 (48.1%)	33 (33.3%)	
Mitral valve surgery			0.197
- No	870 (30.9%)	24 (24.2%)	
- Yes	1950 (69.1%)	75 (75.8%)	
Tricuspid valve surgery			0.000
- No	2406 (85.3%)	69 (69.7%)	
- Yes	414 (14.7%)	30 (30.3%)	
Pacemaker			0.148
- No	2736 (97.0%)	93 (93.9%)	
- Yes	84 (3.0%)	6 (6.1%)	
Acute Myocardial Infarction 48h			1.000
- No	2814 (99.8%)	99 (100.0%)	
- Yes	6 (0.2%)	0 (0.0%)	
Dialysis			0.000
- No	2769 (98.2%)	87 (87.9%)	
- Yes	51 (1.8%)	12 (12.1%)	
Renal failure			0.000
- No	2706 (96.0%)	75 (75.8%)	
- Yes	114 (4.0%)	24 (24.2%)	
High creatinine			0.000
- No	2682 (95.1%)	63 (63.6%)	
- Yes	138 (4.9%)	36 (36.4%)	

Results for bivariate associations with mortality from the MINE analysis, a test used to detect overall associations, indicated that pulmonary hypertension, left atrium size, high creatinine, renal failure, tricuspid procedure and aortic valve procedure were the main unadjusted predictors of mortality according to the Maximal Information Coefficient (Table 5).

**Table 5:** MINE results.

X	Y	MIC
Pulmonary hypertension	Left atrial size	0.15346
High creatinine	Renal failure	0.15128
Tricuspid procedure	Pulmonary hypertension	0.14919
Aortic valve surgery	Left atrial size	0.10897
Aortic valve surgery	Pulmonary hypertension	0.09832
Reoperation	Pulmonary hypertension	0.09128
High creatinine	Dialysis	0.08324
Reoperation	Left atrial size	0.07857
Ejection fraction	Age	0.07731
Pulmonary hypertension	Ejection fraction	0.07690

During our feature engineering, the following variables were deemed as having high near-zero variance frequency ratios and percent uniqueness, and despite their clinical relevance, were eliminated from our final model: emergency surgery, cardiogenic shock, concomitant valve and revascularization procedure, presence of pacemaker, myocardial infarct within 48 hours from the surgical procedure, dialysis, and renal failure. Since the percentage of missing values in our cohort was negligible, we opted for not performing imputation.

**Fig 2:**
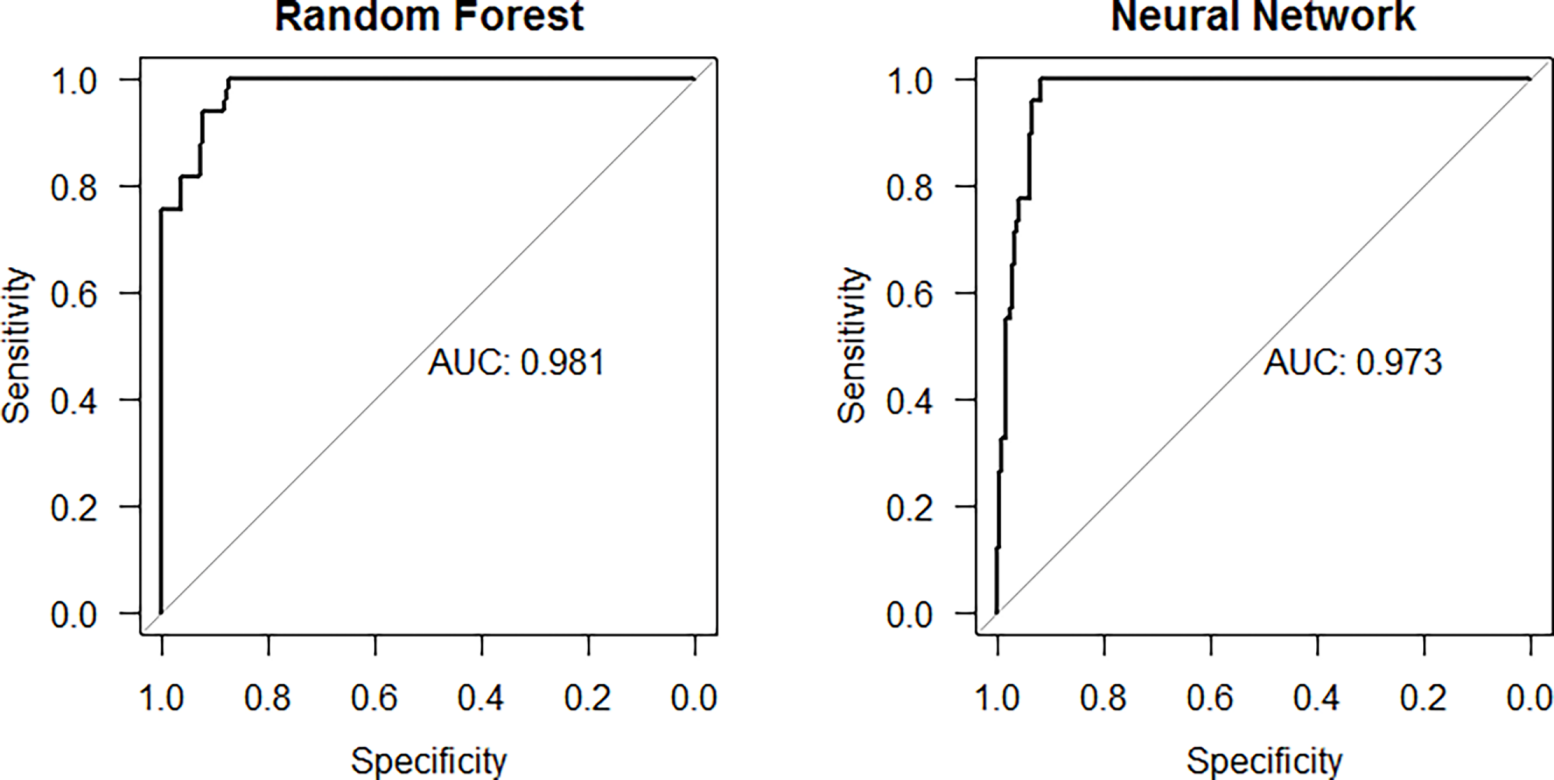
Receiver operating curves compared across models.

Results for all 13 models regarding their overall performance are displayed in Table 6, with the top performing models being Random Forest and Neural Network with areas of 0.982 and 0.952, respectively (Fig 2).

**Table 6:** Performance for all 13 models.

Model	Performance (AUC)	Sensitivity	Specificity
Random Forest	0.982	0.591	1
Neural Network	0.952	0.286	0.994
Support Vector Machines with Radial Basis Function Kernel	0.946	0.347	0.996
Naive Bayes	0.928	0	1
Quadratic Discriminant Analysis	0.919	0.490	0.95
Linear Discriminant Analysis	0.904	0.265	0.967
Nearest Shrunken Centroids	0.903	0	1
Generalized Linear Model	0.890	0.02	0.999
Penalized Logistic Regression	0.890	0.02	0.999
Partial Least Square	0.887	0	1
k-Nearest Neighbors	0.883	0.061	0.997
Mixture Discriminant Analysis	0.850	0.367	0.960
Flexible Discriminant Analysis	0.841	0.347	0.98

**Fig 3:**
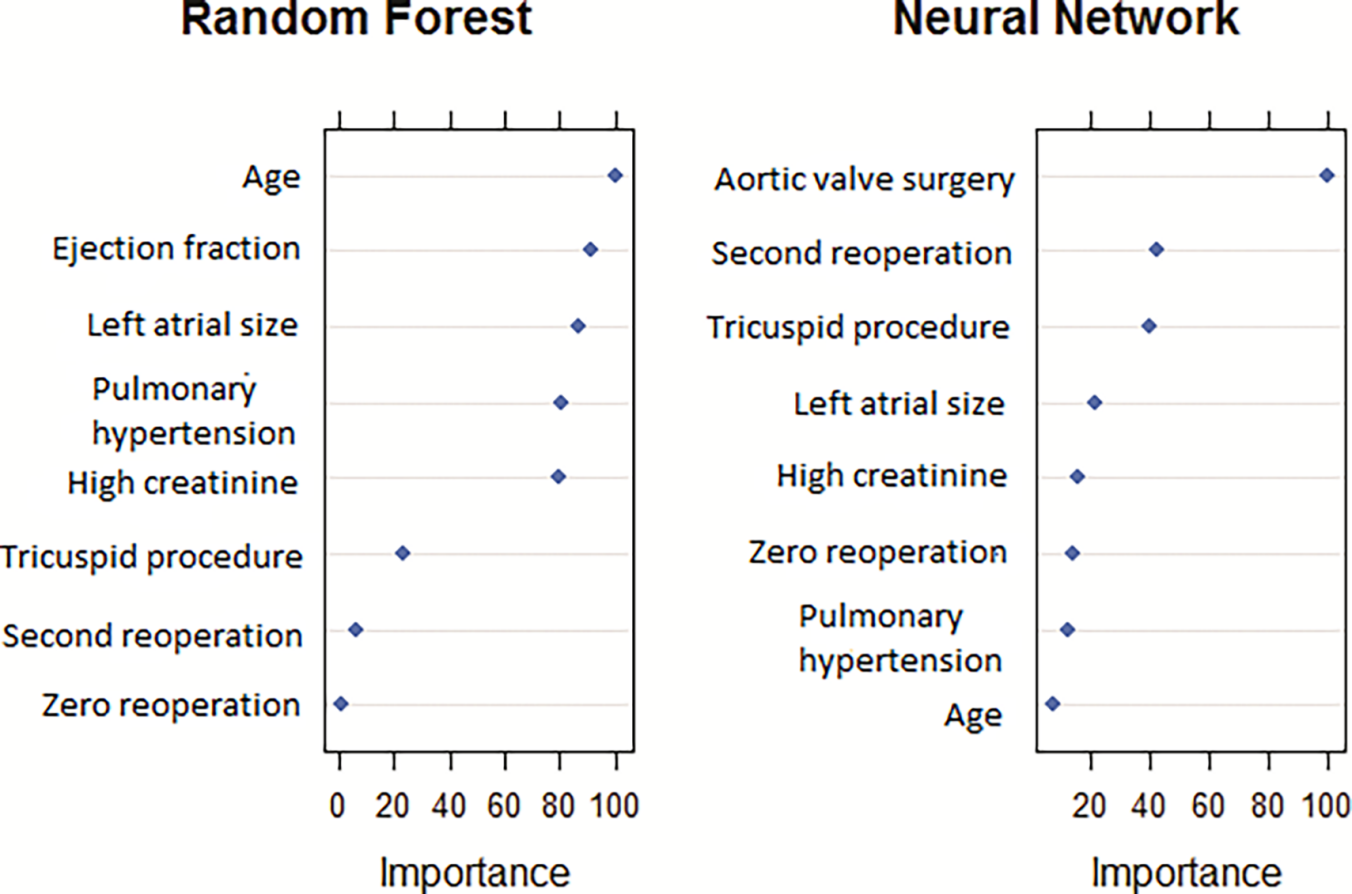
Variable importance grid for the top models.

When evaluating the main predictors among our top two models, we observed that the variables left atrium size, high creatinine, tricuspid procedure, reoperation and pulmonary hypertension were consistently the most influential ones predicting mortality (Fig 3). Comparison across model performance was conducted using an area under the curve, where larger values represent better-combined sensitivity and specificity.

Table 7 summarizes the main finding of our paper by comparing the area under the curve for the best performing RheSCORE model, the 2000 Bernstein-Parsonnet, EuroSCORE II, InsCor, Ambler, Guaragna, and the New York score, demonstrating a substantial improvement in predictive performance in favor of the RheSCORE model making use of Random Forest.

**Table 7:** Area under the curve for the RheSCORE, 2000 Bernstein-Parsonnet, EuroSCORE II, InsCor, Ambler score, Guaragna score and the New York score.

Score	Performance (AUC)
RheSCORE	0.98
2000 Bernstein-Parsonnet	0.876
EuroSCORE II	0.857
InsCor	0.835
Ambler score	0.831
Guaragna score	0.816
New York score	0.834

Finally, we have published a Web application containing the best performing random forest model so that healthcare professionals can calculate predicted mortality rates for individual patients. The application is available at http://www.incor.usp.br/quick/app.html.

## Discussion

To the best of our knowledge, this is the first report of a predictive model specifically designed for patients with rheumatic valve conditions undergoing cardiac procedures, making model results available not as a score but as a Web application. This Web application is promptly available to peers as well as to practitioners at the bedside. We have demonstrated that the RheSCORE model using a random forests algorithm provides a substantially improved predictive performance over previous scores. We also observed that, among the top performing models, the following variables were consistently ranked among the most important in predicting mortality: left atrium size, high creatinine, a tricuspid procedure, a reoperation procedure and the presence of pulmonary hypertension.

We obtained a better prediction performance with the RheSCORE model than with traditional scores; traditional scores have been designed with the intention of being simple to calculate as long as the practitioner could recall their scoring formula at the bedside. Despite their simplicity, efforts to improve the predictive performance of traditional scores have mostly come to a halt in the past decade. Parsonnet [13] was one of the first authors to analyze mortality risk factors in a sample of patients only undergoing coronary artery bypass graft surgery. Eleven years later, a study involving 10,703 patients undergoing coronary artery bypass graft surgery as well as valve procedures in 10 centers in New Jersey (USA) led to the 2000 Bernstein-Parsonnet score [2]. Given that Parsonnet only involved an American-based sample, the EuroSCOREs [3] was subsequently developed with 19,000 patients from 128 European centers, this score later being reformulated to create EuroSCORE II [9]. With scores now validated in both European and American populations, our team validated the 2000 Bernstein-Parsonnet and EuroSCORE scores among a Brazilian group of patients undergoing coronary artery bypass graft surgery and valve procedures, also generating the new InsCor model [5]. Given that the InsCor score was specifically designed to address the needs of a patient population that is essentially different from their American and European counterparts, the InsCor was the most appropriate of all three [4]. Although there are a number of other scores in the literature, to our knowledge none of them has substantially improved prediction performance, including the Ambler score [10] with an area under the curve of 0.77 in the original publication and 0.73 in the Brazilian population [14]. Scores specifically designed for patients undergoing valve procedures have also not achieved substantially greater performance, including Hannan's score [11] with an area under the curve of 0.79. To our knowledge, Hannan's score has not been previously validated in a sample involving patients from developing countries. Finally, Guaragna published a valve-specific model validated in a Brazilian population [12], with a resulting area under the curve of 0.83 in the original publication and 0.78 in a subsequent validation [15]. Our current development of the RheSCORE model can be considered as the next generation in model development, with prediction results that far surpass the ones from classical scores.

Our finding regarding the importance of left atrial size is aligned with previous reports [16], often surpassing the combination of multiple isolated predictors. For example, in one previous series evaluating surgical outcome predictors, left atrial size was found to be the primary outcome predictor, although this association might vanish in the presence of atrial fibrillation [17]. The importance of left atrial size can be explained since this parameter reflects both the severity and duration of mitral regurgitation, both of which can significantly affect mortality risk.

Regarding the predictive importance of high creatinine levels, our findings concur with many previous publications demonstrating its association with high mortality after cardiac surgery when compared with controls. Of importance, previous findings have demonstrated that even small serum creatinine changes after surgery can significantly affect mortality, this association being independent of other well-established perioperative risk indicators [18].

Our results regarding the importance of tricuspid procedures in predicting mortality align with previous publications point to these interventions as the second highest risk for mortality after valvular heart surgery [19]. In a separate series evaluating determinants of surgical mortality after cardiac surgery, the tricuspid procedure was again shown to be the second highest determinant of mortality [20] among a selected group of 19 predictors. Although not evaluated in our study, studies report that mortality rates for re-operated patients undergoing tricuspid procedures can rise by up to 37% [21], a factor that should be taken into account when planning procedures as well as discussing potential risks with patients.

Given the increased surgical trauma as well as the underlying reasons leading to a re-operation, heart valve re-operations are known to be performed with an acceptable operative mortality with some patient categories presenting elevated risks [22] ultimately underscoring the need for appropriate risk prediction and stratification in relation to therapeutic options and preoperative selection.

Age remains an independent predictor of mortality in this population although a lower value is associated with rheumatic patients [23]. Our data indicate that the left ventricular dysfunction, analyzed by LVEF, also is associated with mortality after heart valve surgery in rheumatic patients. Like the previous report on non-specific rheumatic patients [24], the number of valve reoperation was an independent predictor of hospital mortality. Finally, as opposed to previous publications, there was no report of an association between gender and hospital mortality [22]

Despite a significant improvement in predictive performance when compared to previously reported scores, our study does have limitations. First, although our model is transparent enough to point to the most important variables predicting mortality, it does not provide a clear causal path. In other words, our model does not offer an instrument that could help us better design quality improvement programs toward a reduction in mortality rates after cardiac surgery among patients with rheumatic valve conditions. This limitation could be addressed in future studies where causal models such as Bayesian Networks can be used to predict not only mortality but also to determine a clinically interpretable causal model as well as to conduct causal experiments. Second, our cohort of rheumatic patients comes from one of the cities with the highest income in Latin America. This probably explains why the average age of our subjects is in the upper limit of upper-middle-income countries included in the REMEDY Study [25].

## Conclusions

In conclusion, we believe that future studies should further validate the predictive performance of the RheSCORE model among patient populations from other countries, evaluate how healthcare professionals might use our Web application in daily clinical practice, and also investigate how that use might affect their clinical decision making. Despite these pending evaluations, and in view of our results steering to a superior predictive performance, we recommend the incorporation of the RheSCORE model into daily practice when attempting to predict mortality risk among patients undergoing cardiac surgical procedures for rheumatic valve conditions.

### Perspectives

- The RheSCORE model, developed specifically for rheumatic-related conditions, has superior predictive performance when compared to previous traditional scores.
- The most important variables predicting mortality across different models were left atrium size, high creatinine, a tricuspid procedure, a reoperation procedure and the presence of pulmonary hypertension.
- As a model-based mortality prediction tool, the RheSCORE model can be accessed through Web browsers and smartphones at http://www.incor.usp.br/quick/app.html
